# A pivotal role for HOXB7 protein in endocrine resistant breast cancer

**DOI:** 10.18632/oncoscience.263

**Published:** 2015-11-15

**Authors:** Kideok Jin, Saraswati Sukumar

**Affiliations:** ^1^ Breast Cancer Program, Sidney Kimmel Comprehensive Cancer Center, Johns Hopkins University School of Medicine, Baltimore, MD, USA

**Keywords:** HOXB7, breast cancer, tamoxifen-resistance, ER, MYC

## Abstract

HOXB7 is a homeodomain containing transcription factor which plays a pivotal role in tamoxifen resistant breast cancer. Our work has shown that overexpression of HOXB7 renders cells tamoxifen resistant by mobilizing a number of receptor tyrosine kinase pathways. EGFR expression is upregulated by direct binding of HOXB7 to the EGFR promoter, while HOXB7 functions as a cofactor with ERα to cause overexpression of multiple ER-target genes, including HER2, in tamoxifen resistant breast cancer cells. Probing the pathway further, we found that miR-196a and MYC are upstream regulators of HOXB7 expression. Mechanistically, HOXB7 and ERα jointly upregulate HER2 which phosphorylates MYC. Thus stabilized, MYC in turn suppresses miR-196a. Loss of miR-196a results lifts the quelling influence of miR-196a on HOXB7 expression. Besides shedding light on the intricate interplay of events occurring in tamoxifen resistant breast cancer, the work identifies a number of new therapeutic targets capable of restoring sensitivity of breast cancer cells to tamoxifen.

## DISCUSSION

The estrogen receptor status is an important factor in designing treatment of ER-positive breast cancer. About 70% of ER-positive breast cancers show significant response to aromatase inhibitors and selective estrogen receptor modulators (SERMs) such as tamoxifen (TAM). Although tamoxifen is relatively safe and has potent anti-tumor activity initially, one third of women with ER-positive breast cancer undergoing TAM monotherapy are at risk of recurrence due to the development of TAM resistance [1]. Tamoxifen resistance can be classified into two categories: intrinsic or acquired [2]. ER expression is maintained at detectable levels in the majority of the breast tumors with acquired resistance. In these tumors, ER continues to regulate tumor proliferation [3]. Twenty percent of patients who relapse on tamoxifen respond to the pure ER-antagonist, fulvestrant, or to aromatase inhibitors [4, 5]. Long term endocrine therapy is frequently related to upregulation of receptor tyrosine kinases (RTKs) such as EGFR and HER2 as well as the activation of ER to promote endocrine resistant tumor cell proliferation [6], but details of mechanisms of activation of RTKs and ER were not yet clear.

In 2012, Jin et al. [7] showed that HOXB7 acts as a transcriptional factor rendering breast cancers tamoxifen-resistant through direct activation of the receptor tyrosine kinase, EGFR. Inhibition of EGFR activity by gefitinib in MCF-7-HOXB7 cells significantly decreased cellular viability and restored sensitivity to tamoxifen. In addition, HOXB7 enhanced the expression of several EGFR ligands such amphiregulin, heparin-binding-EGF and transforming growth factor-α by cross-talk with an activated ERα signaling pathway. These results implicated HOXB7 as an orchestrator of the function of ERα and EGFR pathways and raised the possibility that high HOXB7 expression could be a significant marker for anti-EGFR therapy in tamoxifen resistant breast cancer.

**Figure 1:**
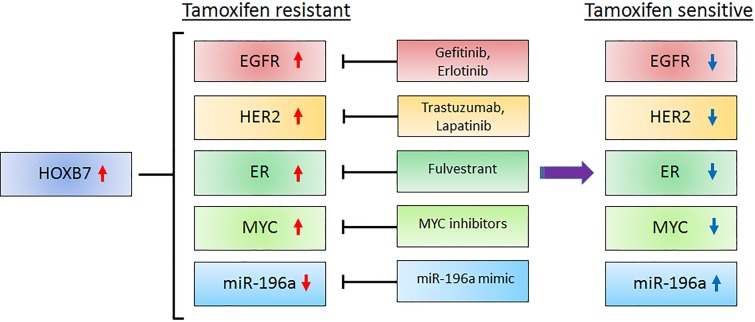
The proposed model of HOXB7-regulated pathways and therapeutic strategies to overcome tamoxifen resistance in breast cancer

Given the robust upregulation of ER activity by HOXB7, we explored the role of HOXB7 in activated ER-mediated tamoxifen resistance. In a recent manuscript Jin et al. [8] demonstrated that HOXB7 acts as an ERα cofactor thereby enhancing the expression of ER-target genes including HER2, and conferred tamoxifen resistance to breast cancer cells. HOXB7 physically interacts with ERα and promotes upregulation of ER-target genes. Using HOXB7 ChIP analysis of known ER-binding sites in ER-target genes, their work revealed that the ER–HOXB7 complex directly binds to ER binding sites at the target gene locus to enhance transcriptional activity. Moreover, the HOXB7-ER complex recruits ER cofactors (AIB1, SRC-1, CBP, p300, NCOR, and PAX2), and HOXB7 cofactors (PBX2 and MEIS1) to the ER-binding site. These events occur by the formation of a chromatin loop between the ER-binding site and HOXB7-binding sites as shown by the chromosome conformation capture (3C) assay. Thus, HOXB7 as a key coactivator in ER genomic function enhances the expression of ER target genes. In addition, they showed that the expression of HER2 is upregulated by the HOXB7-ER complex directly. Exploring how HOXB7 expression is controlled in breast cancer cells, miR-196a was identified as an upstream controller of HOXB7 expression. As predicted, miR-196a overexpression in tamoxifen resistant MCF-7 cells reduced HOXB7 and resensitized cells to tamoxifen, while depletion of miR-196a in parental MCF-7 cells resulted in tamoxifen resistance. Furthermore, the loop was further extended by showing that stabilized MYC (phosphorylated by high HER2 signaling in tamoxifen resistant cells) repressed miR-196a expression leading to an increase in HOXB7 levels and consequently, enhanced tamoxifen resistance (Figure 1). These findings of the interactions and cross talk between HOXB7, HER2 and MYC bore clinical relevance, since high expression of the three molecules in primary breast cancer is predictive of endocrine resistance in patients with ER-positive breast cancer. These findings could have therapeutic implications since combination treatment of tumor xenografts of tamoxifen resistant breast cancer cells, BT474, with a MYC inhibitor (10058-F4) and HER2 inhibitor (Trastuzumab) significantly reduced tumor growth in immunodeficient NSG mice (Figure 1).

Taken together, these studies on HOXB7 provide significant insights and knowledge of pathways critical to the development of tamoxifen resistant breast cancer. The findings also imply that the EGFR signaling pathway and feedback loop of MYC-HOXB7-HER2 could serve as therapeutic targets to prevent tamoxifen resistance.
